# Improvement in Health-Related Quality of Life After Community Acquired Pediatric Septic Shock

**DOI:** 10.3389/fped.2021.675374

**Published:** 2021-08-18

**Authors:** Neethi P. Pinto, Robert A. Berg, Athena F. Zuppa, Christopher J. Newth, Murray M. Pollack, Kathleen L. Meert, Mark W. Hall, Michael Quasney, Anil Sapru, Joseph A. Carcillo, Patrick S. McQuillen, Peter M. Mourani, Ranjit S. Chima, Richard Holubkov, Vinay M. Nadkarni, Ron W. Reeder, Jerry J. Zimmerman, the Life After Pediatric Sepsis Evaluation (LAPSE) Investigators

**Affiliations:** ^1^Department of Anesthesiology and Critical Care Medicine, Children's Hospital of Philadelphia, Philadelphia, PA, United States; ^2^Department of Anesthesiology and Critical Care Medicine, Children's Hospital of Los Angeles, University of Southern California, Los Angeles, CA, United States; ^3^Department of Pediatrics, Children's National Health System, Washington, DC, United States; ^4^Department of Pediatrics, Children's Hospital of Michigan, Central Michigan University, Detroit, MI, United States; ^5^Department of Pediatrics, Nationwide Children's Hospital, Columbus, OH, United States; ^6^Department of Pediatrics, C.S. Mott Children's Hospital, University of Michigan, Ann Arbor, MI, United States; ^7^Department of Pediatrics, Mattel Children's Hospital, University of California, Los Angeles, Los Angeles, CA, United States; ^8^Department of Critical Care Medicine, Children's Hospital of Pittsburgh, University of Pittsburgh Medical Center, Pittsburgh, PA, United States; ^9^Department of Pediatrics, Benioff Children's Hospital, University of California, San Francisco, San Francisco, CA, United States; ^10^Department of Pediatrics, University of Colorado School of Medicine and Children's Hospital of Colorado, Aurora, CO, United States; ^11^Division of Critical Care Medicine, Department of Pediatrics, University of Cincinnati College of Medicine, Cincinnati Children's Hospital Medical Center, Cincinnati, OH, United States; ^12^Department of Pediatrics, University of Utah, Salt Lake City, UT, United States; ^13^Department of Pediatrics, Seattle Children's Hospital, Seattle Research Institute, University of Washington School of Medicine, Seattle, WA, United States

**Keywords:** health-related quality of life, pediatric sepsis, survivorship, long-term outcomes, pediatric critical care, PICU, pediatric critical illness

## Abstract

**Background:** Although some pediatric sepsis survivors experience worsening health-related quality of life (HRQL), many return to their pre-illness HRQL. Whether children can improve beyond baseline is not known. We examined a cohort of pediatric sepsis survivors to determine if those with baseline HRQL scores below the population mean could exhibit ≥10% improvement and evaluated factors associated with improvement.

**Methods:** In this secondary analysis of the Life After Pediatric Sepsis Evaluation prospective study, children aged 1 month to 18 years admitted to 12 academic PICUs in the United States with community-acquired septic shock who survived to 3 months and had baseline HRQL scores ≤ 80 (i.e., excluding those with good baseline HRQL to allow for potential improvement) were included. HRQL was measured using the Pediatric Quality of Life Inventory or Stein-Jessop Functional Status Scale.

**Findings:** One hundred and seventeen children were eligible. Sixty-one (52%) had ≥ 10% improvement in HRQL by 3 months. Lower pre-sepsis HRQL was associated with increased odds of improvement at 3 months [aOR = 1.08, 95% CI (1.04–1.11), *p* < 0.001] and 12 months [OR = 1.05, 95% CI (1.02–1.11), *p* = 0.005]. Improvement in HRQL was most prevalent at 3 month follow-up; at 12 month follow-up, improvement was more sustained among children without severe developmental delay compared to children with severe developmental delay.

**Interpretation:** More than half of these children with community acquired septic shock experienced at least a 10% improvement in HRQL from baseline to 3 months. Children with severe developmental delay did not sustain this improvement at 12 month follow-up.

## Introduction

All-cause mortality after pediatric critical illness has declined over the past 4 decades with recently reported mortality rates of 2.4% in the United States (1). However, in-hospital mortality does not completely capture the extent of the impact of pediatric critical illness (2). Indeed, children who survive critical illness remain medically vulnerable with evidence of increasing rates of morbidity and mortality after discharge from the hospital through 3 year follow-up (3).

This phenomenon is also apparent among the subset of critically ill children who experience sepsis. In this setting, in-hospital mortality rates are 5–10% in developed nations, but the risk of morbidity and longer term mortality among survivors remain high (4). Both clinicians and families identify survival and health related quality of life (HRQL) to be the most important outcomes after pediatric critical illness with research increasingly focusing on these patient-centered outcomes (5).

Characterization of the trajectory of morbidity and mortality and examination of the associated critical illness risk factors for death and persistent, serious HRQL disability among pediatric sepsis survivors were the objectives of Life After Pediatric Sepsis Evaluation (LAPSE) investigation (1R01HD073362) (4, 6). Consistent with other studies suggesting long-term serious post-sepsis morbidity (7), 37% of the LAPSE cohort experienced a decrease in HRQL from baseline pre-sepsis at 3 months, and 35% remained below their baseline HRQL at 12 months (4). Yet, most survivors recovered to baseline HRQL. In this secondary analysis, we examined whether a subset of these children could not only recover but exhibit improvement in their quality of life. Secondarily, we determined risk factors associated with lack of improvement, providing essential information to guide future development of post-PICU interventions to optimize patient outcomes. We hypothesized that >10% of these children would not only recover but exhibit a ≥10% improvement from baseline pre-sepsis HRQL scores by 3 month follow-up, presuming that ICU and post-ICU medical interventions could effectively treat their pre-existing diseases and/or pathophysiologic processes. The primary objective of this study was to quantify the frequency of ≥10% HRQL improvement and evaluate the factors associated with this degree of improvement 3 months after sepsis in a secondary analysis of the LAPSE dataset. In addition, we evaluated the factors associated with ≥10% improvement after 12 months and described the trajectory of HRQL improvement from 3 to 12 months post-sepsis in these children.

## Materials and Methods

### Design and Setting

This study is a secondary analysis of the LAPSE prospective cohort investigation. Twelve academic pediatric intensive care units (PICUs) in the United States recruited children for the primary study between January 1, 2014 through June 30, 2017. Institutional review boards across all study sites approved the study. Written permission was obtained from parents of all study participants.

### Participants

Children were included in the LAPSE study if they were between the ages of 44 weeks gestation and 18 years, had documented or suspected sepsis or infection, and met at least 2 of the 4 criteria for systemic inflammatory response syndrome (including abnormal leukocyte count/differential and/or abnormal body temperature), cardiovascular organ dysfunction necessitating fluid resuscitation and vasoactive-inotropic infusion, and pulmonary organ dysfunction requiring invasive or non-invasive pressure support or mechanical ventilation that was initiated within 72 h of hospital admission and within 48 h of PICU admission. Children were excluded if any of the following conditions applied: unable to be enrolled within 48 h of admission or participate in long term follow up, previously enrolled, wards of the state, limitations of care including do not resuscitate (DNR) orders, thermal or electrical burns, or guardians/parents unable to speak English or Spanish. Further details of inclusion and exclusion criteria have been previously published (4, 6). In the primary study, 389 children were included; LAPSE subjects were included in this secondary analysis if they survived to 3 months and had complete baseline and 3 month HRQL data. LAPSE subjects were excluded if their baseline HRQL scores were >80 [i.e., excluding children with good baseline HRQL (8)], because discerning substantial post-sepsis HRQL improvement in children with good baseline pre-sepsis HRQL is limited due to ceiling effects (9) (i.e., children with good HRQL at baseline pre-sepsis cannot attain substantial improvement in HRQL at 3 months post-sepsis). So, we focused *a priori* on patients with HRQL scores ≤ 80, a score 20% below the maximum Pediatric Quality of Life inventory (PedsQL™) (10) or Stein Jessop Functional Status Score (FSII-R) (11). Importantly, the longitudinal validity of the PedsQL has been demonstrated, suggesting that exclusion of children with good baseline pre-sepsis HRQL would not result in a bias toward improved HRQL among the population over time (12, 13). Since diagnosis-specific information was not available at the patient level in this dataset, we utilized absence of pre-sepsis severe developmental delay (per parental assessment) as a proxy for childhood conditions with greater potential for HRQL improvement beyond baseline pre-sepsis.

### Patient and Hospitalization Characteristics

Patient demographic data and data related to severity of illness [using Pediatric Risk of Mortality (PRISM) (14) and Pediatric Logistic Organ Dysfunction (PELOD-2) (15) scores], chronic comorbidities [using the Pediatric Medical Complexity Algorithm (PMCA) (16) and data from the Pediatric Health Information System^1^], immunodeficiency status, and baseline function [using Pediatric Cerebral Performance Category (PCPC) (17), Pediatric Overall Performance Category (POPC) (17), and the Functional Status Scale (FSS) (18)] were collected at study enrollment. Pre-sepsis global presence or absence of severe developmental delay was obtained from parental report at study enrollment. The Ten-Item Personality Inventory (19) was utilized to assess personality traits for children aged ≥14 years. Serial assessment of family functioning was achieved utilizing the Family Assessment Device (20), and parental psychosocial status was assessed utilizing the Brief Symptom Inventory (21, 22). Clinical data including PELOD-2 scores, occurrence of cardiac arrest, requirement of ICU related therapies (vasoactive inotropic medications, mechanical ventilation, extracorporeal life support, red cell transfusion, or renal replacement therapy), neurologic insult, and PICU and hospital lengths of stay were obtained from the electronic health record. Neurologic insult was defined as: (1) change in neurologic examination including anisocoria, pathologic breathing pattern, stereotypic or flaccid posture, autonomic storming, (2) seizure activity and/or abnormal electroencephalogram, (3) new anoxic-ischemic injury on brain imaging, or (4) treatment for increased intracranial pressure. Chronic comorbid conditions were defined by PMCA designations as: (1) significant chronic physical, mental, or developmental conditions expected to last at least a year affecting ≥ 2 body systems requiring health care resource utilization above the level needed for a healthy child and treatment/control of the condition and with the potential to be episodically or continuously debilitating; (2) progressive conditions associated with health deterioration and decreased life expectancy in adulthood; or (3) conditions requiring technology dependence for ≥ 6 months (16). These patient and hospitalization data were examined for association with improvement in HRQL following pediatric septic shock.

### Health Related Quality of Life Outcomes

Parents of children who did not have severe developmental delay (per parental assessment) completed the PedsQL™, and parents of children with severely delayed development (per parental assessment) completed the FSII-R at study entry to determine the baseline pre-sepsis HRQL status (4, 6, 23). The term “baseline pre-sepsis” reflects the child's baseline status prior to admission for sepsis. Parents subsequently completed serial HRQL assessments at 3 and 12 months following PICU admission. Both instruments are validated, reliable, and internally consistent (10, 11). All scores are reported on a 0–100 scale with higher scores representing better HRQL. The minimal clinically important difference (MCID) is 4.5 points for the PedsQL™ (10). The primary outcome was a 10% improvement in HRQL, because it is a substantial improvement and is well-beyond the MCID for the PedsQL™. Although an MCID has not been established for the FSII-R, we used the same percentage increase from individual baseline FSII-R scores to define improved HRQL.

### Statistical Analysis

Patient and hospitalization characteristics were summarized. The association of these characteristics with an improvement in HRQL from baseline to 3 months and baseline to 12 months was evaluated. Associations were evaluated with Fisher's exact-test for categorical variables and the Wilcoxon rank-sum test for continuous variables. Summary statistics reported are counts and percentages [*n* (%)] for categorical variables and median (Q1, Q3) for continuous variables.

In order to further assess predictors of improved HRQL at 3 months in children, logistic regression models were created. Variables at least marginally associated with improvement in univariable modeling (*p* < 0.20) were considered as candidates for inclusion in multivariable modeling if available for ≥ 90% of the cohort. A multivariable model was created using bidirectional stepwise selection with a criterion of *p* < 0.20 to enter and *p* < 0.05 to stay in the final model. This analytic approach was also taken to investigate predictors of improved HRQL at 12 months. This same approach was taken to investigate predictors of improved HRQL at 3 and 12 months in children with and without severe developmental delay by parental report. Analyses were performed using SAS 9.4 (SAS Institute; Cary, NC). *P*-values reported were based on two-sided alternatives and considered significant when < 0.05.

The primary analysis of this study was analyzed using a complete case analysis. However, because there was considerable loss to follow-up at 12 months, and more severe cases and worse outcomes at hospital discharge were associated with increased loss to follow-up, a complete case analysis showing the trajectory of quality of life over time would be significantly biased. Multiple imputation techniques were used with the intent to reduce this bias and to present HRQL trajectory from baseline pre-sepsis to the end of study follow-up 12 months later. Imputed datasets were created using a sequence of regression models in IVEware to account for correlations between variables. Imputed datasets contained the observed data along with data drawn from a posterior predictive distribution replacing missing values. This approach allowed us to more fully utilize the observed data while theoretically reducing bias.

Details regarding the imputation methods and statistical software used can be found in previously published LAPSE articles (4, 6). A notable difference is that a more robust number of multiple imputed data sets, *n* = 50, were generated for this investigation.

## Results

**Figure 1:**
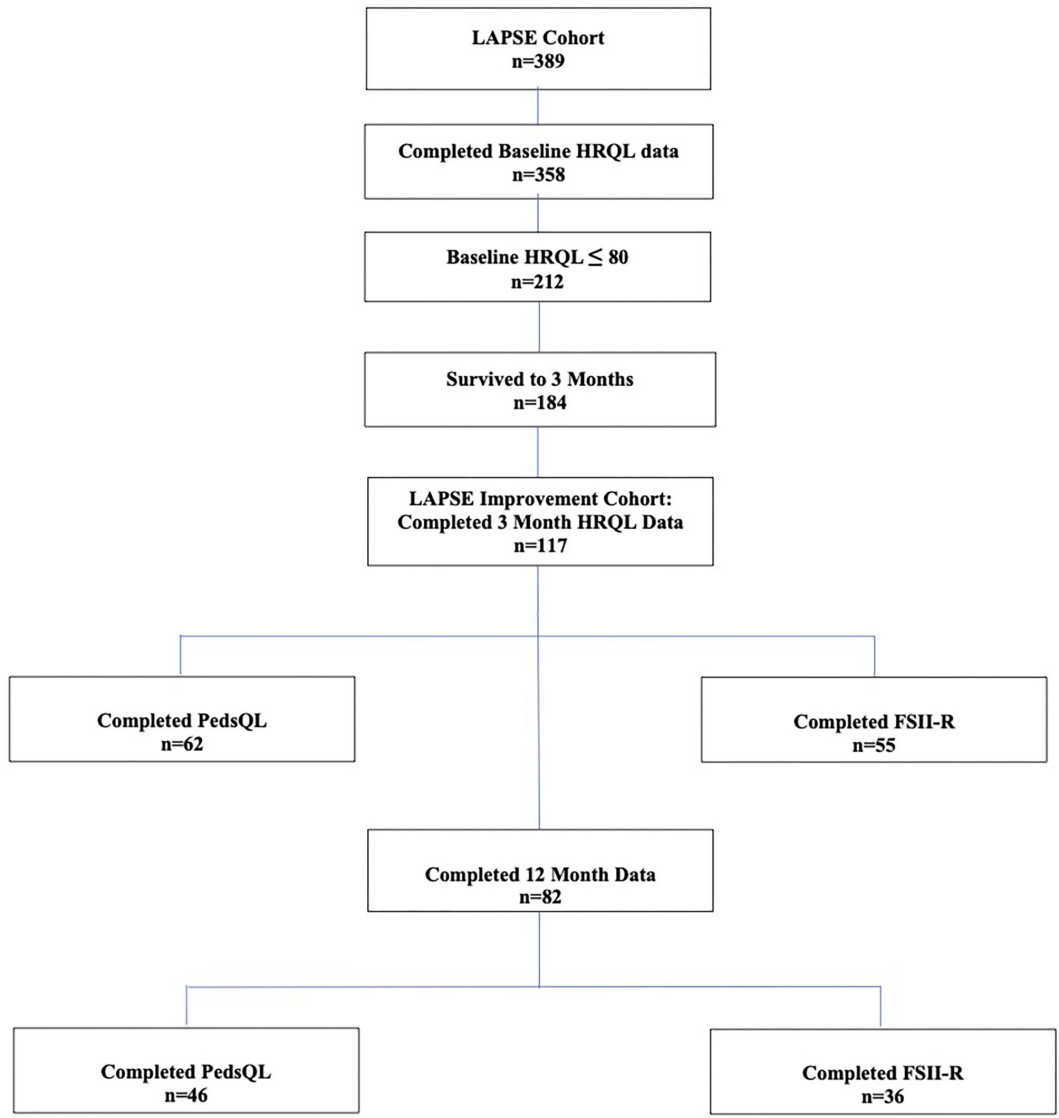
Study Flow Diagram.

Among the 358 children from the primary LAPSE cohort who had complete baseline pre-sepsis HRQL data, 212 (59%) had a baseline pre-sepsis HRQL score ≤ 80; 184 survived to 3 months; and 117 of these children had complete 3 month data and were eligible for inclusion in this study (Figure 1). Overall, 62 completed PedsQL™, and 55 completed FSII-R. On average, children were aged 6.7 years and 46% female (Table 1). The majority of children had a complex medical condition (68%) and some degree of disability (78%). Baseline pre-sepsis HRQL scores were 64.3 (55.4, 75.0) [median (Q1, Q3)]. PRISM and PELOD scores were 10 (6, 16) and 8 (6, 11), and PICU duration of stay was 7.9 (5.0, 12.2) days.

**Table 1:** Patient and hospitalization characteristics at baseline.

**Patient characteristics**	**Overall (*N* = 117)**
**Age** (years), median (Q1, Q3)	6.7 (2.0, 12.7)
**Female gender**, *n* (%)	54 (46.2%)
**Medical complexity algorithm classification**, *n* (%)	
None/non-complex	38 (32.5%)
Complex	79 (67.5%)
**Baseline pre-sepsis HRQL**, PedsQL™ or FSII-R, median (Q1, Q3)	64.3 (55.4, 75.0)
**Baseline functional assessments**	
**POPC score**, *n* (%)	
Normal/good	26 (22.2%)
Mild disability	24 (20.5%)
Moderate disability	29 (24.8%)
Severe disability	38 (32.5%)
**Functional status scale**, median (Q1, Q3)	9.0 (6.0, 13.0)
**Guardian education level**, *n* (%)	
High school grad, GED, or less	32 (27.4%)
Vocational school, some college, or 2 year degree	37 (31.6%)
College degree	30 (25.6%)
Graduate or doctoral degree	16 (13.7%)
Unknown	2 (1.7%)
**Annual household income**, *n* (%)	
Unknown	8 (6.8%)
Prefer not to answer	6 (5.1%)
< $30,000	30 (25.6%)
$30,000 to < $50,000	24 (20.5%)
$50,000–100,000	22 (18.8%)
$100,000 or more	27 (23.1%)
**Immunocompromised status**, *n* (%)	21 (17.9%)
**Hospitalization summaries**	
PRISM score, median (Q1, Q3)	10.0 (6.0, 16.0)
**Nature of infection at time of eligibility**, ***n*****(%)**	
Documented (culture, PCR)	56 (47.9%)
No documented infection	61 (52.1%)
PELOD, First day of PICU admission, median (Q1, Q3)	8.0 (6.0, 11.0)
Sum of PELOD, median (Q1, Q3)	45.0 (27.0, 74.0)
Sum of vasoactive-inotropic score, median (Q1, Q3)	28.5 (8.0, 61.5)
Duration of mechanical ventilation (days), median (Q1, Q3)	7.0 (4.0, 12.0)
Renal replacement therapy, *n* (%)	7 (6.0%)
Renal replacement duration (days), median (Q1, Q3)	0.0 (0.0, 0.0)
Cardiopulmonary arrest or chest compressions, *n* (%)	4 (3.4%)
Extracorporeal life support, *n* (%)	4 (3.4%)
PICU length of stay (days), median (Q1, Q3)	7.9 (5.0, 12.2)
Hospital length of stay (days), median (Q1, Q3)	15.0 (8.8, 25.5)
Neurologic insult(s) during PICU stay, *n* (%)	48 (41.0%)
Overall brief symptom inventory score (Day 28), median (Q1, Q3)	6.0 (1.0, 15.0)
Family assessment device score (Day 28), median (Q1, Q3)	1.6 (1.2, 2.0)

*PedsQL™, Pediatric Quality of Life Inventory; FSII-R, Stein-Jessop Functional Status Scale; GED, general educational development (high-school equivalency test); PELOD, Pediatric logistic organ dysfunction; PCR, polymerase chain reaction*.

Sixty-one children (52%) exhibited at least 10% improvement from pre-sepsis HRQL at 3 months. Children who improved and those who did not improve by at least 10% were not different with regard to age, sex, guardian education level, annual household income, medical complexity, pre-existing disability, functional status, or immunocompetence status (Table 2). Similarly, they did not differ with regard to severity of illness, need for intensive care unit-related therapies, PICU or hospital duration of stay, or occurrence of neurologic insult experienced during PICU stay. Notably, children with at least 10% HRQL improvement had a lower baseline pre-sepsis HRQL compared to those who did not improve, 60.7 (50.0, 69.2) vs. 70.5 (62.5, 75.9), *p* < 0.001. Parents of children whose HRQL improved had a lower overall Brief Symptom Inventory scale at day 28, 3.0 (0.0, 10.0) vs. 8.0 (3.0, 18.0), *p* = 0.02.

**Table 2:** Patient and hospitalization characteristics by improvement status at 3 and 12 months.

**Patient characteristics**	**3 months**			**12 months**		
	**No improvement** **(*n* = 56)**	**Improved by ≥ 10%** **(*n* = 61)**	***p*-value**	**No improvement** **(*n* =43)**	**Improved by ≥ 10%** **(*n* = 39)**	***p*-value**
Age (years), *n* (%)	7.4 (2.4, 11.8)	6.3 (1.8, 12.7)	0.567^a^	5.7 (1.8, 12.7)	7.6 (2.4, 14.1)	0.373^a^
Female, *n* (%)	26 (46.4%)	28 (45.9%)	1.000^b^	26 (60.5%)	15 (38.5%)	0.076^b^
Medical complexity algorithm classification, *n* (%)			0.433^b^			1.000^b^
None/non-complex	16 (28.6%)	22 (36.1%)		16 (37.2%)	15 (38.5%)	
Complex	40 (71.4%)	39 (63.9%)		27 (62.8%)	24 (61.5%)	
Baseline pre-sepsis PedsQL™/FSII-R (*N* = 117), median (Q1, Q3)	70.5 (62.5, 75.9)	60.7 (50.0, 69.2)	<0.001^a^	71.4 (59.8, 76.1)	60.7 (50.0, 73.1)	0.014^a^
Baseline POPC score, *n* (%)			0.897^a^			0.810^a^
Normal/good	12 (21.4%)	14 (23.0%)		11 (25.6%)	11 (28.2%)	
Mild disability	13 (23.2%)	11 (18.0%)		9 (20.9%)	9 (23.1%)	
Moderate disability	13 (23.2%)	16 (26.2%)		10 (23.3%)	7 (17.9%)	
Severe disability	18 (32.1%)	20 (32.8%)		13 (30.2%)	12 (30.8%)	
Baseline Functional Status Scale, median (Q1, Q3)	8.0 (6.0, 13.0)	10.0 (6.0, 13.0)	0.862^a^	9.0 (6.0, 12.0)	9.0 (6.0, 13.0)	0.706^a^
Guardian education level, *n* (%)			0.088^b^			0.331^b^
High school grad, GED, or less	11 (19.6%)	21 (34.4%)		7 (16.3%)	12 (30.8%)	
Vocational school, some college, or 2 year degree	23 (41.1%)	14 (23.0%)		13 (30.2%)	10 (25.6%)	
College degree	12 (21.4%)	18 (29.5%)		13 (30.2%)	13 (33.3%)	
Graduate or doctoral degree	9 (16.1%)	7 (11.5%)		9 (20.9%)	4 (10.3%)	
Annual household income, *n* (%)			0.219^b^			0.198^b^
< $30,000	16 (28.6%)	14 (23.0%)		9 (20.9%)	7 (17.9%)	
$30,000 to < $50,000	8 (14.3%)	16 (26.2%)		6 (14.0%)	11 (28.2%)	
$50,000–100,000	14 (25.0%)	8 (13.1%)		11 (25.6%)	5 (12.8%)	
$100,000 or more	14 (25.0%)	13 (21.3%)		15 (34.9%)	8 (20.5%)	
Immunocompromised status, *n* (%)	12 (21.4%)	9 (14.8%)	0.470^b^	9 (20.9%)	7 (17.9%)	0.786^b^
**Hospitalization summaries**						
Overall PRISM score, median (Q1, Q3)	9.5 (6.0, 17.0)	11.0 (7.0, 15.0)	0.523^a^	8.0 (5.0, 16.0)	12.0 (6.0, 15.0)	0.285^a^
Nature of infection at time of eligibility, *n* (%)			0.854^b^			0.657^b^
Documented (culture, PCR)	26 (46.4%)	30 (49.2%)		25 (58.1%)	20 (51.3%)	
No infection documented	30 (53.6%)	31 (50.8%)		18 (41.9%)	19 (48.7%)	
PELOD, First day of PICU admission, median (Q1, Q3)	8.0 (5.5, 11.0)	8.0 (6.0, 10.0)	0.939^a^	8.0 (6.0, 11.0)	7.0 (5.0, 11.0)	0.472^a^
Sum of PELOD, median (Q1, Q3)	51.5 (24.0, 95.0)	40.0 (29.0, 67.0)	0.497^a^	44.0 (29.0, 94.0)	40.0 (23.0, 74.0)	0.370^a^
Sum of Vasoactive-Inotropic Score, median (Q1, Q3)	26.0 (7.8, 79.8)	29.0 (8.0, 57.5)	0.772^a^	29.0 (8.0, 61.0)	30.0 (10.0, 57.0)	0.813^a^
Duration of mechanical ventilation (days), median (Q1, Q3)	6.5 (4.0, 16.5)	7.0 (4.0, 10.0)	0.615^a^	7.0 (4.0, 12.0)	7.0 (2.0, 11.0)	0.415^a^
Renal replacement therapy, *n* (%)	4 (7.1%)	3 (4.9%)	0.708^b^	3 (7.0%)	3 (7.7%)	1.000^b^
Cardiopulmonary arrest or chest compressions, *n* (%)	3 (5.4%)	1 (1.6%)	0.348^b^	1 (2.3%)	1 (2.6%)	1.000^b^
Extracorporeal life support, *n* (%)	0 (0.0%)	4 (6.6%)	0.120^b^	1 (2.3%)	2 (5.1%)	0.602^b^
PICU length of stay (days) , median (Q1, Q3)	8.7 (4.3, 16.2)	7.9 (5.3, 11.0)	0.745^a^	7.9 (5.1, 12.9)	6.8 (3.6, 11.1)	0.559^a^
Hospital length of stay (days) , median (Q1, Q3)	15.9 (8.0, 29.3)	14.8 (9.5, 21.2)	0.629^a^	16.9 (9.2, 26.7)	14.9 (9.2, 23.4)	0.676^a^
Neurologic insult(s) during PICU stay, *n* (%)	23 (41.1%)	25 (41.0%)	1.000^b^	24 (55.8%)	14 (35.9%)	0.081^b^
Overall BSI score (Day 28), median (Q1, Q3)	8.0 (3.0, 18.0)	3.0 (0.0, 10.0)	0.020^a^	6.5 (1.0, 18.0)	4.0 (0.0, 10.0)	0.199^a^
Family assessment device score (Day 28), median (Q1, Q3)	1.7 (1.2, 2.0)	1.5 (1.2, 1.8)	0.131^a^	1.6 (1.1, 1.9)	1.7 (1.2, 2.0)	0.457^a^

*PedsQL™, Pediatric Quality of Life Inventory; FSII-R, Stein-Jessop Functional Status Scale; GED, general educational development (high-school equivalency test); PELOD, Pediatric logistic organ dysfunction; BSI, Brief Symptom Inventory*.

a *Wilcoxon rank-sum-test*.

b *Fishers Exact-test*.

Among the 117 children, 82 children had HRQL data (46 completed PedsQL™ and 36 completed FSII-R) available at 12 months. Of these, 39 children (48%) demonstrated at least 10% improvement in HRQL from their baseline pre-sepsis (Table 2). Children who exhibited at least a 10% HRQL improvement at 12 months had a lower baseline HRQL [60.7 (50.0, 73.1)] compared to children who did not improve at 12 months [71.4 (59.8, 76.1), *p* = 0.014] (Table 2).

In adjusted analyses, higher baseline HRQL (PedsQL™ or FSII-R) was associated with decreased odds of improvement in HRQL by 10% at 3 months [aOR = 0.93, 95% CI (0.90–0.96), *p* < 0.001] or in other words, for every unit decrease in HRQL at baseline, the odds of improved HRQL increased by 8% [aOR = 1.08, 95% CI (1.04–1.11), *p* < 0.001] (Table 3). Higher baseline pre-sepsis HRQL was also associated with a decreased odds of improved HRQL among children with available data at 12 months [aOR = 0.95 (0.90–0.98), *p* = 0.005] (Table 3). That is, for every unit decrease in HRQL at baseline, the odds of improved HRQL increased by 5% [aOR = 1.05, 95%CI (1.02–1.1), *p* = 0.005].

**Table 3:** Multivariable analyses of improved HRQL.

	**Improved HRQL in study** **cohort**		**Improved HRQL in children** **without severe** **developmental delay**		**Improved HRQL in children** **with severe** **developmental delay**	
	**Adjusted odds ratio** **(95% CI)**	***p*-value**	**Adjusted odds ratio** **(95% CI)**	***p*-value**	**Adjusted odds ratio** **(95% CI)**	***p*-value**
**At 3 months**	*N* = 117		*N* = 62		*N* = 55	
Total baseline HRQL^*^ score	0.93 (0.90–0.96)	<0.0001	0.94 (0.89–0.98)	0.005	0.92 (0.86–0.97)	0.002
PICU length of stay (days)	–	–	–	–	0.94 (0.88–0.99)	0.024
**At 12 months**	*N* = 82		*N* = 46		*N* = 36	
Total baseline HRQL^*^ score	0.95 (0.90–0.98)	0.005	–	–	0.89 (0.80–0.97)	0.005
Neurologic insult(s) during PICU stay	0.34 (0.12–0.88)	0.025	0.28 (0.08–0.93)	0.037	–	–
Female	–	–		–	0.08 (0.01–0.44)	0.003

* *HRQL score was PedsQL™ for Children Without Severe Developmental Delay and FSII-R for Children With Severe Developmental Delay*. *–Not significant in univariable analysis*.

Assuming that severe developmental delay would limit potential for HRQL improvement, we compared children with and without severe developmental delay per parental assessment. For children without severe developmental delay, higher baseline PedsQL™ was associated with lower odds of improved HRQL at 3 months [aOR = 0.94, 95% CI (0.89–0.98), *p* = 0.005] or in other words, for every unit decrease in baseline PedsQL™ the odds of improved PedsQL™ increased by 6% [aOR = 1.06, 95% CI (1.02–1.12), *p* = 0.005]. Lack of neurologic insult during PICU stay was associated with an increased odds of improved HRQL at 12 months [aOR = 3.57, 95% CI (1.1–15.5), *p* = 0.037] for children without severe developmental delay. Among children with severe developmental delay, lower baseline FSII-R and shorter PICU duration of stay were associated with an increased odds of improved HRQL at 3 months [aOR = 1.09, 95% CI (1.03–1.16), *p* = 0.002 and aOR = 1.06, 95% CI (1.01–1.14), *p* = 0.024, respectively]. Lower baseline FSII-R and male sex were both associated with an increased odds of improved HRQL at 12 months [aOR = 1.12, 95% CI (1.03–1.25), *p* = 0.005 and aOR = 12.5, 95% CI (2.3–100), *p* = 0.003, respectively] for children with severe developmental delay.

**Figure 2:**
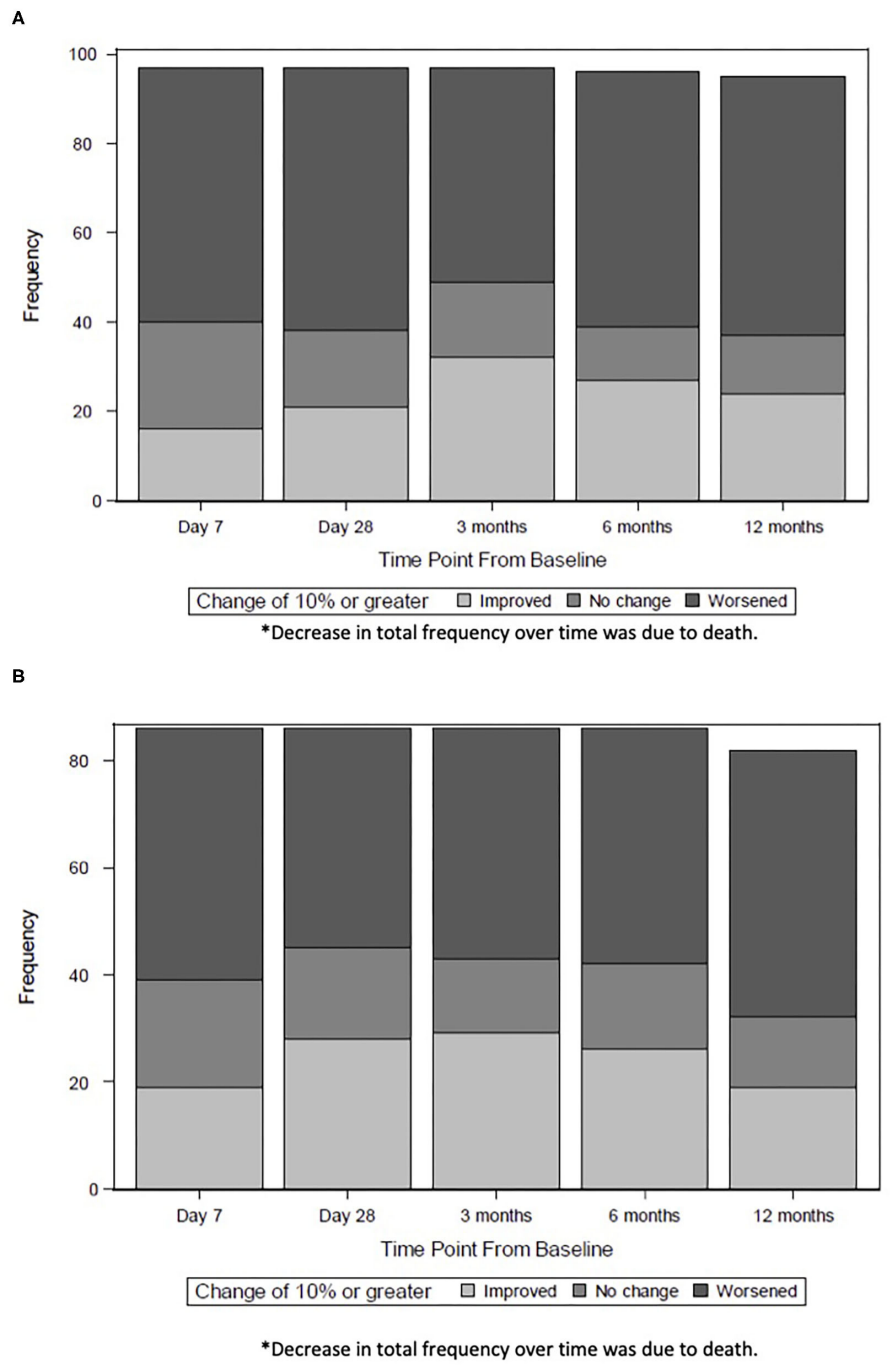
**(A)** Trajectory of HRQL For Children without Severe Developmental Delay*. The number of children without severe developmental delay who exhibited at least 10% improvement in HRQL peaked at 3 months (32 children) and decreased by 12 months (23 children). **(B)** Trajectory of HRQL for Children with Severe Developmental Delay*. The number of children without severe developmental delay who exhibited at least 10% improvement in HRQL peaked at 3 months (29 children) and decreased by 12 months (16 children). *Decrease in total frequency over time was due to death.

We also examined the trajectory of HRQL from baseline pre-sepsis to 12 months after sepsis, using multiple imputation to account for children with missing data. The number of children who exhibited at least 10% improvement in HRQL peaked by 3 months. By 12 months, fewer children maintained this degree of improvement, particularly among those with severe developmental delay (Figures 2A,B).

## Discussion

Consistent with our hypothesis, our data demonstrate that a substantial number of patients with community acquired septic shock admitted to a PICU exhibit improvement in HRQL. Specifically, 52% of children with baseline pre-sepsis HRQL scores at least 20% below maximal scores experienced a ≥10% improvement in HRQL from their baseline pre-sepsis to 3 months. A lower pre-sepsis quality of life was associated with increased likelihood of 10% improvement in HRQL at 3 and 12 month follow-up. Improvement in HRQL was most notable at 3 months; children without preexisting severe developmental delay sustained mild improvement during the first year of follow-up whereas those with preexisting severe developmental delay did not.

These data demonstrate that more than half of these pediatric sepsis survivors recovered and exhibited improvement in HRQL by ≥10% above baseline pre-sepsis at 3 months, an increase substantially greater than the MCID. Our analyses also reveal that 48% of these children continued to have ≥10% improvement in HRQL at 12 months. In an era where 90–95% of children with septic shock survive hospitalization, our data demonstrate that not only do children survive but many are resilient and some thrive.

Over the last couple decades, many pediatric and adult post-sepsis investigations have described ongoing morbidity, focusing on the Post-Intensive Care Syndrome (4, 7, 24, 25). In contrast, we hypothesized that >10% of the LAPSE cohort would have ≥10% improvement in HRQL above baseline pre-sepsis. Surprisingly, we found a much larger number of children with such improvement, and we identified factors associated with this degree of improvement. Baseline pre-sepsis HRQL was the factor most strongly associated with ≥10% improvement in HRQL, as lower pre-sepsis quality of life was associated with increased odds of improved HRQL at 3 and 12 months. One possible reason for this finding is that detecting a 10% HRQL improvement among children with pre-sepsis HRQL in the almost “good” range is limited by ceiling effects (9). Another possibility is that children with higher pre-sepsis HRQL were presumed to be more nearly normal, and were thus not discharged with the same degree of support or medical follow-up as children who have a lower pre-sepsis HRQL. Alternatively, these children (or their parents as proxy reporters) may exhibit HRQL response shifts which have been described in cancer survivors and epilepsy patients (26, 27). These shifts are changes in self-response that occur over time due to recalibration, reconceptualization, or reprioritization rather than due to true physiologic change (28). Parents of children with high baseline HRQL and parents of children with low baseline HRQL may experience varying degrees of response shifts and may differ in how they define good HRQL after a life threatening event.

We identified additional factors associated with improvement above baseline pre-sepsis HRQL during the first year after critical illness. Among the overall study cohort and children without severe developmental delay, neurologic insults during PICU stay were associated with a decreased odds of improved HRQL at 12 months, consistent with findings from other studies (29). This finding is intuitive and highlights the lasting sequelae of critical illness 12 months after PICU admission. Among children with severe developmental delay, decreased PICU duration of stay and male sex were associated with an increased odds of improved HRQL above baseline pre-sepsis at 3 and 12 months, respectively. Presumably, duration of stay is a proxy for illness acuity, severity of illness, physical and psychologic deconditioning, and/or overall impact of critical illness. The association of male sex with increased odds of improvement is unexpected, particularly considering that other high-risk pediatric populations (e.g., preterm infants and infants with congenital heart disease) have more favorable outcomes among girls (30, 31).

Notably, the trajectories of improvement for children diverge after peaking at 3 months. The patterns of recovery seen in our study suggest that more children without severe delayed development may exhibit sustained recovery compared to children with severe developmental delay. Children with severe developmental delay were presumably more susceptible to new medical problems or progression of their underlying disease or comorbid conditions. Consistent with our findings, Polic et al. described children with initial recovery in HRQL at 6 months after PICU admission, followed by relative plateaus by 12 months post-discharge (32). Similarly, other investigators found that children may exhibit a rapid rate of recovery up to 3 months post-discharge, but functional status deteriorates in some children over time (3, 29). Our data highlight that short durations of follow-up through 3 months of discharge may be falsely reassuring and not adequately representative of longer-term outcomes, particularly for children with severe developmental delay.

There were important limitations to our study. First, these patients were treated at large children's hospitals in the US, limiting generalizability to other populations. Second, we experienced 30% loss to follow-up at 12 months which may have introduced bias if the children lost to follow up differed from our study population. We used multiple imputation with the intent to reduce this bias for our HRQL trajectory analyses. However, our regression models did not use imputed datasets and reflect observed data only. Our data reflect the inherent difficulty associated with long-term PICU cohorts after discharge despite attempts to mitigate these logistical challenges, including monetary incentivization for participation (4). Expanding this study to include more diverse geographic regions and a larger sample size would further inform our understanding of the trajectory of HRQL after pediatric sepsis. Third, diagnosis-specific data and information regarding acute comorbid conditions were not available at the individual patient level precluding our ability to provide a physiologic rationale for why some of these children improved beyond baseline pre-sepsis HRQL other than the possibilities of effective therapies for pre-existing diseases or pathophysiologic processes. Although our cohort was similar to the overall cohort (which included children with higher baseline HRQL) with regard to age, gender, baseline PRISM and PELOD scores, immunocompromised status, duration of mechanical ventilation, and hospital length of stay, we did not have information regarding other factors such as specific co-morbid diseases, socioeconomic status, or access to health care that may have contributed to the observed improvement in HRQL. As such, we are unable to attribute the observed improvement in HRQL primarily to services rendered in the intensive care unit. Fourth, presence or absence of severe developmental delay was determined by parental assessment introducing a potential selection bias. However, the functional status scores (POPC and FSS) for children whose parents reported severely delayed development were consistent with severe disability or moderate to severe physical dysfunction, suggesting accurate categorization of developmental status by these parents. Fifth, use of parent-proxy report on the HRQL instruments is another potential source of bias. Nevertheless, patient-report among critically ill septic children is not practical as these children are typically limited in their capacity to provide self-reports (due to intubation, sedation, developmental delay, or young age), and parent-proxy report has been validated for the PedsQL (33).

In conclusion, more than half of children with community acquired septic shock and baseline pre-sepsis HRQL scores at least 20% below maximum scores experienced at least a 10% improvement in HRQL from baseline pre-sepsis to 3 months. Lower baseline HRQL was associated with an increased odds of improved HRQL at 3 and 12 months in these children. Improvement in HRQL was most prevalent at 3 month follow-up; by 12 month follow-up, fewer children with severe developmental delay exhibited sustained improvement compared to children without severe developmental delay. Our data underscore the importance of following children longitudinally after PICU admission.

## Data Availability Statement

The original contributions presented in the study are included in the article/supplementary materials, further inquiries can be directed to the corresponding author.

## Ethics Statement

The studies involving human participants were reviewed and approved by Institutional Review Boards at each participating site: Children's Hospital of Los Angeles, Children's Hospital of Philadelphia, Mott Children's Hospital, St. Louis Children's Hospital, Colorado Children's Hospital, Nationwide Children's Hospital, Children's Hospital of Michigan, Mattel Children's Hospital, Benioff Children's Hospital, National Children's Hospital, Cincinnati Children's Hospital, Pittsburgh Children's Hospital. Written informed consent to participate in this study was provided by the participants' legal guardian/next of kin.

## Author Contributions

NP, RB, RR, and JZ contributed to conception and design of the study. RR and RB performed the statistical analysis. NP wrote the first draft of the manuscript. JZ, RR, and RB wrote sections of the manuscript. All authors contributed to manuscript revision, read, and approved the submitted version.

## Conflict of Interest

JC was funded by the NICHD and NIGMS. MH serves as a paid consultant to LaJolla Pharmaceuticals, and he receives licensing income from Kiadis. Both relationships are unrelated to the content of the current manuscript. RH serves on Data Safety Monitoring Boards for Pfizer, Inc. CN has had financial relationships with Philips Research North America, Hamilton Medical AG, and Nihon Kohden America. JZ has received compensation for the following activities: salary distribution from the University of Washington Medical Group and the Society of Critical Care Medicine (2018–2019 only); royalty payments from Elsevier Publishing for serving as Co-Editor for Pediatric Critical Care; research funding from the National Institutes of Health, Patient-Centered Outcomes Research Institute, Biomedical Advanced Research and Development Authority, and Immunexpress, Seattle; and travel reimbursement to attend board meetings from the Society of Critical Care Medicine. The remaining authors declare that the research was conducted in the absence of any commercial or financial relationships that could be construed as a potential conflict of interest.

## Publisher's Note

All claims expressed in this article are solely those of the authors and do not necessarily represent those of their affiliated organizations, or those of the publisher, the editors and the reviewers. Any product that may be evaluated in this article, or claim that may be made by its manufacturer, is not guaranteed or endorsed by the publisher.

## Footnotes

^1^https://www.childrenshospitals.org/phis.

## Life After Pediatric Sepsis Evaluation (LAPSE)

Russell Banks, MS, Department of Pediatrics, University of Utah, Salt Lake City, UT, United States

Whitney Coleman, MRA, BSN, Department of Pediatrics, University of Utah, Salt Lake City, UT, United States

Wren Haaland, MPH, Department of Pediatrics, Seattle Children's Hospital, Seattle Research Institute, University of Washington School of Medicine, Seattle, WA, United States

Julie McGalliard, BA, Department of Pediatrics, Seattle Children's Hospital, Seattle Research Institute, University of Washington School of Medicine, Seattle, WA, United States

Samuel Sorenson, BS, Department of Pediatrics, University of Utah, Salt Lake City, UT, United States

James W. Varni, PhD, Department of Pediatrics, Texas A&M University, College Station, TX, United States

David Wessel, MD, Department of Pediatrics, Children's National Health System, Washington, DC, United States

Kathryn Whitlock, MPH, Department of Pediatrics, Seattle Children's Hospital, Seattle Research Institute, University of Washington School of Medicine, Seattle, WA, United States

## Abbreviations

HRQL: Health-related quality of life
LAPSE: Life After Pediatric Sepsis Evaluation
PedsQL™: Pediatric Quality of Life inventory
FSII-R: Stein Jessop Functional Status Score
PICU: Pediatric Intensive Care Unit
PRISM: Pediatric Risk of Mortality
PELOD-2: Pediatric Logistic Organ Dysfunction
MCID: Minimal Clinically Important Difference.
